# Characteristics and determinants of endurance cycle ergometry and six-minute walk distance in patients with COPD

**DOI:** 10.1186/1471-2466-14-97

**Published:** 2014-05-31

**Authors:** Vasileios Andrianopoulos, Scott S Wagers, Miriam TJ Groenen, Lowie E Vanfleteren, Frits ME Franssen, Frank WJM Smeenk, Ioannis Vogiatzis, Emiel F M Wouters, Martijn A Spruit

**Affiliations:** 1 Department of Research & Education, CIRO+, Centre of Expertise for Chronic Organ Failure, Hornerheide 1, 6085 NM Horn, Eindhoven, Netherlands; 2 BioSci Consulting, Weg naar Geneuth 95, 3631, Maasmechelen, Limburg, Belgium; 3 Department of Respiratory Medicine, Catharina Hospital, Eindhoven, Netherlands; 4 Department of Physical Education and Sport Sciences, National and Kapodistrian University of Athens, Ethnikis Antistasis 41, 17237 Athens, Greece; 5 Institute of Clinical Exercise and Health Sciences, University of the West of Scotland, Paisley PA1 2BE, Scotland, United Kingdom; 6 Department of Respiratory Medicine, Maastricht University Medical Centre (MUMC+), P.Debyelaan 25, 6202 AZ Maastricht, Netherlands

**Keywords:** Exercise, 6MWT, CET, CPET, COPD

## Abstract

**Background:**

Exercise tolerance can be assessed by the cycle endurance test (CET) and six-minute walk test (6MWT) in patients with Chronic Obstructive Pulmonary Disease (COPD). We sought to investigate the characteristics of functional exercise performance and determinants of the CET and 6MWT in a large clinical cohort of COPD patients.

**Methods:**

A dataset of 2053 COPD patients (43% female, age: 66.9 ± 9.5 years, FEV_1_% predicted: 48.2 ± 23.2) was analyzed retrospectively. Patients underwent, amongst others, respiratory function evaluation; medical tests and questionnaires, one maximal incremental cycle test where peak work rate was determined and two functional exercise tests: a CET at 75% of peak work rate and 6MWT. A stepwise multiple linear regression was used to assess determinants.

**Results:**

On average, patients had impaired exercise tolerance (peak work rate: 56 ± 27% predicted, 6MWT: 69 ± 17% predicted). A total of 2002 patients had CET time of duration (CET-T_end_) less than 20 min while only 51 (2.5%) of the patients achieved 20 min of CET-T_end_ . In former patients, the percent of predicted peak work rate achieved differed significantly between men (48 ± 21% predicted) and women (67 ± 31% predicted). In contrast, CET-T_end_ was longer in men (286 ± 174 s *vs* 250 ± 153 s, p < 0.001). Also, six minute walking distance (6MWD) was higher in men compared to women, both in absolute terms as in percent of predicted (443 m, 67%predicted vs 431 m, 72%predicted, p < 0.05). Gender was associated with the CET-T_end_ but BMI, FEV_1_ and FRC were related to the 6MWD highlighting the different determinants of exercise performance between CET and 6MWT.

**Conclusions:**

CET-T_end_ is a valuable outcome of CET as it is related to multiple clinical aspects of disease severity in COPD. Gender difference should temper the interpretation of CET.

## Background

Constant work rate cycle ergometry, the so-called cycle endurance test (CET), has been put forth as an important assessment of exercise tolerance for patients with chronic obstructive pulmonary disease (COPD) [1-3]. CET endurance time (CET-T_end_) can be used in the evaluation of exercise tolerance [4]. Yet our understanding of CET lags behind that of the commonly used six minute walk test (6MWT) [5-8]. In fact, the first report of minimal clinically important difference for the CET has only been published in 2008 [9].

Neder and colleagues [10], in a detailed study aimed at plotting power duration relationship from a series CETs, found that the average critical power asymptote (i.e.: the power at which an individual can cycle for 20 minutes) in patients with COPD was 81% of the peak work rate achieved on a maximal incremental test. Anecdotal clinical experience, however, suggests that few patients with COPD can cycle that long, even at 75% of peak work rate, that is the level at which most CETs are performed [11]. CET performed at this fixed proportion of peak incremental workload capacity does not cause identical physiological stress among patients [12]. Furthermore, 20 minutes is considered as the maximal allowable CET-T_end_ for a CET [13], and the test is usually stopped at that point.

Constant work rate endurance protocols in CET are based on the maintenance of exercise effort until the exhaustion [14]. Although CET is considered as submaximal exercise, it is aimed to assess the maximal aerobic capacity of patients and it is suggested as a highly repeatable test across run-in visits [15]. However, the utility of CETs at 75% peak work rate comes into doubt as more patients would cycle to the obligatory stopping point of 20 minutes [10]. Similarly, the 6MWT even though self-paced, it can be considered as a maximal sustainable exercise in patients with COPD [16]. Both testing modalities for assessing exercise tolerance may provoke different physiological responses and cause different levels of dyspnea and fatigue during the exertion to patients with COPD [17,18]. Furthermore, the sex of patients has been related to dyspnea in the 6MWT [19] and may also determine the exertion during the CET. From a physiological point of view, CET and 6MWT may not be interchangeable exercise tests and the determinants of exercise performance between the tests can differ.

Herein, we report a retrospective analysis of CET-T_end_ in a large clinical cohort of patients with COPD undergoing initial evaluation for pulmonary rehabilitation (PR) [20]. We sought to compare the results of CETs in a large clinical cohort to those of a 6MWT and we sought to determine how often a clinical cohort of patients with COPD achieved a maximal CET-T_end_ of 20 minutes during a CET performed at a predetermined 75% of peak work rate. Moreover, we investigated the determinants of CET-T_end_ and 6MWD.

## Methods

### Patients

We extracted data from the records of 2991 patients with the diagnosis of COPD who were evaluated during the initial assessment of a comprehensive pulmonary rehabilitation program at CIRO+, center of expertise for chronic organ failure in Horn (The Netherlands) between January 1, 2005 and July 1, 2013 [20]. Of these records, 2053 patients met the following inclusion criteria: all necessary data present, a primary diagnosis of COPD, a FEV_1_/FVC ratio of ≤0.70, evidence that they had reached a physiological maximum during the incremental maximal cardiopulmonary exercise test on a stationary cycle ergometer (i.e., ventilation ≥85% of maximal voluntary ventilation; heart rate ≥80% of the age-predicted heart rate; symptom score for dyspnea ≥7/10 points; and/or symptoms score for fatigue ≥7/10 points), CET done at 75% of peak work rate, and not representing a repeat admission for the same patient. Ethical approval was not indicated because all of the tests were done as part of the routine initial assessment, and analyzed retrospectively. The Board of Directors of CIRO + approved the use of de-identified patients’ record.

### Testing

As part of routine initial assessment, patients underwent, amongst other tests and questionnaires, a maximal incremental cycle test where peak work rate is determined. Subsequently, on a different day, patients performed a CET at 75% of the determined peak work rate. Patients also performed two 6MWTs [21] while the test with the longest distance was used for further analysis. All tests were done according to ATS/ERS guidelines [1,8]. Routine post-bronchodilator spirometry, physical examination, medical history, and Medical Research Council (MRC) dyspnea scale were obtained. Using dual energy x-ray absorptiometry (DEXA), the fat free mass index (FFMI) for each individual was calculated.

### Statistics

All analyses were carried out using SPSS 19, Graphpad Prism 6 and Sigmaplot 12. Data are reported as means with standard deviation (±SD). For parametric data, comparison between men and women were made using a two tailed unpaired t-test, and for categorical data Pearson Chi-Square tests were used, as appropriate. Frequency distributions were used to determine performance quartiles for the CET-T_end_ and six-minute walk distance (6MWD) allowing for comparison of the clinical parameters between quartiles. For all comparisons, patients reaching CET-T_end_ of 20 minutes (51 patients, 2.5%) were excluded from the dataset of 2053 patients as they represented an artificial stopping point, and thereby could artificially reduce standard error. After excluding this group of patients, a dataset of 2002 patients was analyzed for detecting significant differences. For parametric data quartiles were compared using one way ANOVA and for non parametric data quartiles were compared using Kruskal-Wallis test. All multiple comparisons were followed by Bonferonni correction. A stepwise multiple linear regression was used to determine clinical parameters that independently influenced test performance. The level of significance was set at p <0.05.

## Results

### Clinical parameters

On average, patients (n = 2002; CET_end_ <20 min) had moderate to very severe COPD, a normal BMI, normal resting arterial oxygen and carbon dioxide tension, and symptoms of dyspnea. Gender differences were noticed: men were older, and had a significantly higher height, weight, BMI, FFMI, and FEV_1_% predicted compared to women. Then again, women had a significantly worse DLCO. One out of five patients was using long-term oxygen therapy (LTOT) (Table 1).

**Table 1:** Demographics of COPD patients

**Characteristics**	**All patients (n = 2002)**		**Males (n = 1134) (57%)**		**Females (n = 868) (43%)**	
Age, yrs	66.9	(9.5)	69.0	(9.1)	64.3	(9.2)*
Weight, kg	71.9	(16.5)	77.2	(15.9)	65.0	(14.7)*
Height, m	1.68	(0.9)	1.73	(0.7)	1.62	(0.6)*
BMI, kg/m^2^	25.4	(5.2)	25.8	(4.9)	25.0	(5.5)*
FFMI, kg/m^2^	17.0	(2.4)	18.1	(2.2)	15.5	(1.8)*
FEV_1_, % predicted	48.2	(23.2)	47.2	(20.7)	49.6	(26.1)*
FVC, % predicted	95.9	(22.3)	93.0	(21.7)	99.7	(22.7)*
FEV_1_/FVC, r	39.8	(11.8)	38.9	(11.9)	40.9	(11.4)*
FRC, % predicted	150.1	(35.9)	146.3	(37.5)	154.9	(33.0)*
DLCO, % predicted	52.9	(18.1)	55.3	(30.1)	50.9	(25.7)*
PO_2_, kPa	9.8	(2.3)	9.8	(2.2)	9.7	(2.4)
PCO_2_, kPa	5.3	(1.4)	5.2	(1.2)	5.4	(1.5)
MRC dyspnea grade	3.3	(1.1)	3.3	(1.1)	3.4	(1.0)
GOLD stage I	96	(4.8)	48	(4.2)	48	(5.5)
GOLD stage II	649	(32.4)	362	(31.9)	287	(33.1)
GOLD stage III	730	(36.5)	419	(36.9)	311	(35.8)
GOLD stage IV	527	(26.3)	305	(26.9)	222	(25.6)
Receiving LTOT, n, (%)	408	(20.4)	230	(20.3)	178	(20.5)

Average values are shown for exercise tests and clinical parameters. The values are reported for the entire cohort and then for men and women. Asterisk denotes significant differences (p < 0.05).

### Maximal incremental cycle tests

On average, patients had a limited maximal exercise tolerance. The percent of predicted peak work rate achieved was significantly different between men (48 ± 21% predicted) and women (67 ± 31% predicted; p < 0.001; Table 2).

**Table 2:** Parameters of exercise tests in patients with COPD

**Parameters**	**All patients (n = 2002)**		**Males (n = 1134) (57%)**		**Females (n = 868) (43%)**	
**Maximal incremental cycle test**						
Work Rate peak, w	69	(30)	77	(33)	60	(23)**
Work Rate peak, % predicted	56	(27)	48	(21)	67	(31)**
VE, % Max Voluntary Ventilation	87	(24)	86	(23)	89	(25)
HR max, % age-predicted	81	(12)	80	(13)	81	(12)
Systolic BP post, mm Hg	176	(33)	176	(34)	176	(32)
Diastolic BP post, mm Hg	84	(15)	84	(15)	84	(14)
Dyspnea (Borg) post, points	7.2	(2.0)	7.1	(2.0)	7.2	(2.0)
Leg Fatigue (Borg) post, points	5.9	(2.6)	5.8	(2.5)	6.0	(2.6)
**Cycle endurance test (CET)**						
Work Rate mean, w	52	(23)	58	(25)	45	(17)**
Endurance cycling time, s	271	(166)	286	(174)	250	(153)**
Dyspnea (Borg) post, points	5.3	(2.3)	5.4	(2.3)	5.1	(2.3)*
Leg Fatigue (Borg) post, points	5.7	(2.2)	5.8	(2.2)	5.6	(2.1)
**Six-minute walk test (6MWT)**						
6MWD, m	438	(113)	443	(120)	431	(104)*
6MWD, % predicted	69.0	(16.5)	66.9	(17.1)	71.7	(15.3)**
Dyspnea (Borg) post, points	5.2	(4.4)	5.2	(4.2)	5.1	(4.7)
Leg Fatigue (Borg) post, points	4.4	(4.6)	4.4	(4.2)	4.4	(4.8)

Average values are shown for exercise tests and clinical parameters. The values are reported for the entire cohort and then for men and women. Asterisks denotes significant differences (* p < 0.05; ** p < 0.001).

### Endurance tests

Average CET-T_end_ was 271±166 s. This differed significantly between men (286 s) and women (250 s; p < 0.001). Average 6MWD was 438±113 m. The 6MWD was higher in men (443 m) compared to women (431 m; p = 0.019). Then again, 6MWD% predicted differed between men (67 ± 17% predicted) and women (72 ± 15% predicted; p < 0.001; Table 2).

### Distribution of cycle endurance times and six minute walking distances

**Figure 1:**
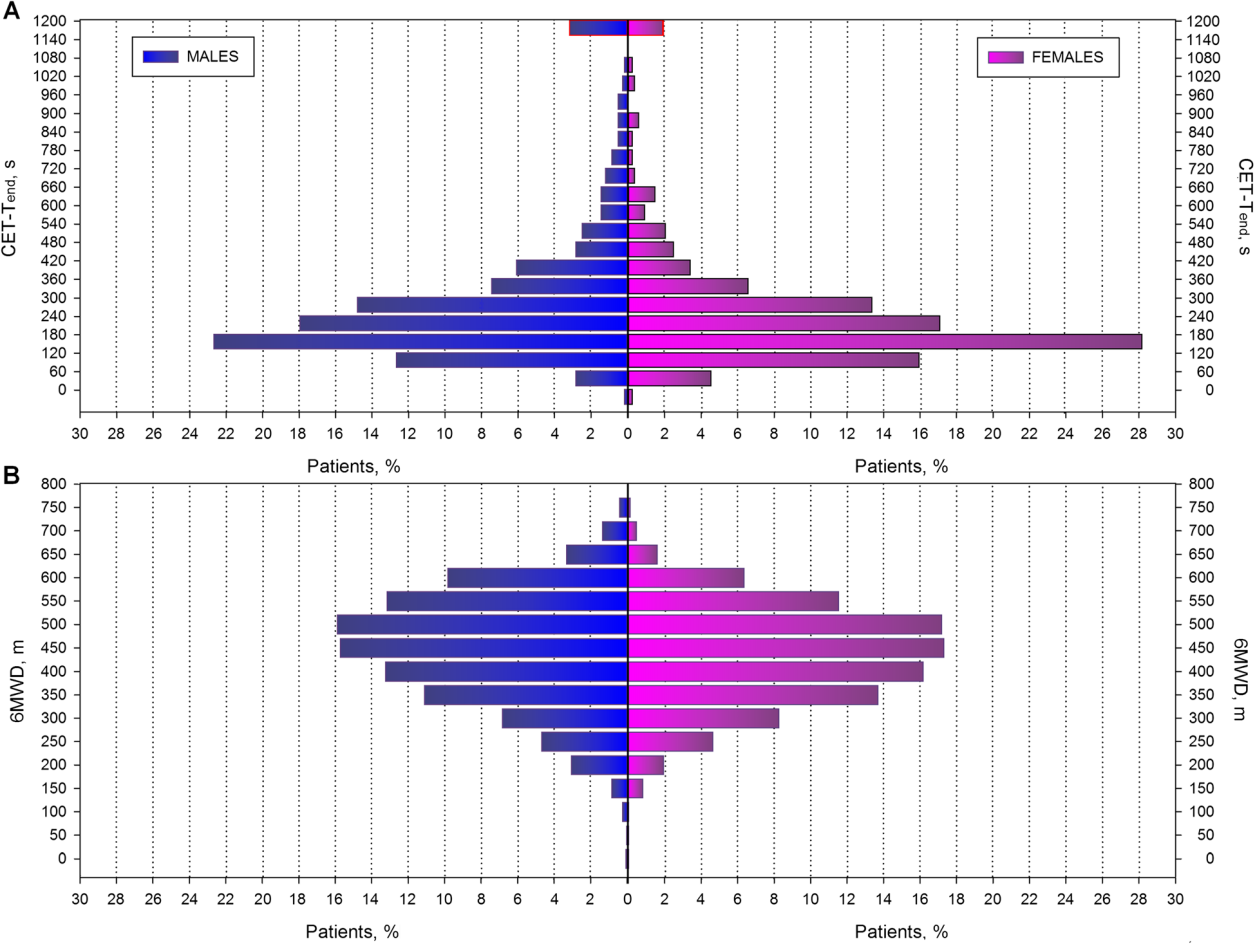
**Frequency distribution of CET-Tend and 6MWD in patients with COPD. (A)** Frequency distribution of 75% of peak work rate CET-T_end._ The number of individuals for each bin of duration time is represented by the height of each column (y-axis). Each bin represents 1 minute (x-axis). Only 51 (2.5%) of the 2053 patients who met the inclusion criteria reached the 20 minute maximal duration. **(B)** frequency distribution of six minute walking distance (6MWD). The number of individuals for each bin of walking meters is represented by the height of each column (y-axis). Each bin represents 1 minute (x-axis).

When separated into 1 minute intervals, the frequency distribution curve for CET-T_end_ shows a leftward kurtosis in men and women (Figure 1A). Only 51, or 2.5%, of patients achieved CET-T_end_ of 20 minutes. When separated into 50 meters intervals, the frequency distribution curve for 6MWD shows a rightward kurtosis in men and women (Figure 1B).

### Associations

**Figure 2:**
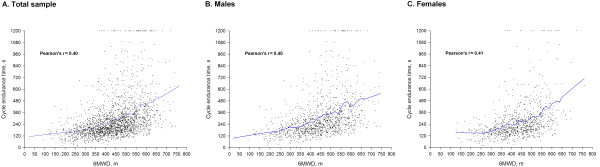
**Pearson’s correlations between CET-T**_**end **_**and 6MWD.** A positive moderate correlation was detected in all models between the CET-T_end_ and 6MWD for all patients and stratified by gender.

The CET-T_end_ and the 6MWD were significantly associated in the whole sample, as well as after stratification for gender presenting a moderate positive correlation (Figure 2).

### Examination of performance quartiles

**Figure 3:**
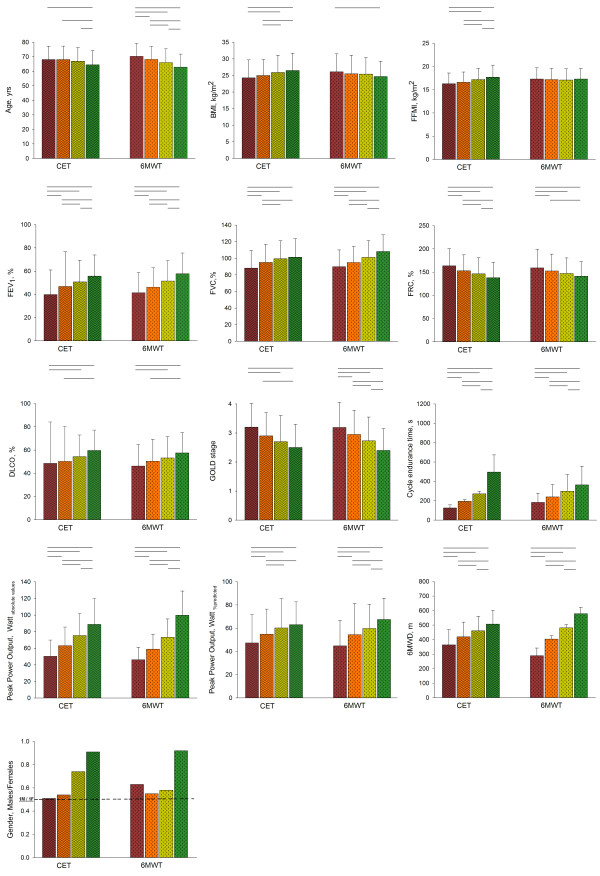
**Performance quartile comparisons.** Using the frequency distribution patients were separated into performance quartiles. With the quartiles increasing left to right (CET: quartiles with wave hashing, 6MWT: quartiles with diagonal hashing). Comparisons were then made between the quartiles of CET and between quartiles of 6MWT for all variables. Significant differences are demonstrated by lines above the relevant bars. Dashed line on gender graph represents the point where is an equal number of men and women.

There is clear performance quartile gradation for FEV_1_%, FVC%, CET-T_end_, peak power output of absolute watt and watt% predicted (sole exception 3^rd^/4^th^ quartile in CET) and the 6MWD with significant differences amongst all quartiles for the exercise performance of CET and the 6MWT (Figure 3). Moreover, there is performance quartile gradation for age with significant differences of age for the 4^th^ and the rest of quartiles for CET and amongst all the quartiles for the 6MWT. Additionally, performance quartile gradation significant differences were detected across the GOLD stages between 1^st^ and 4^th^, 1^st^ and 3^rd^, and 2^nd^ and 4^th^ in cycling and amongst all quartiles in 6MWT. The performance quartile gradation for the FRC demonstrated significant differences in all quartiles for CET while a similar pattern was observed for the 6MWT. The performance quartile gradation for the DLCO demonstrated significant differences amongst 1^st^ and 3^rd^-4^th^, 2^nd^ and 4^th^ quartiles for the CET and 6MWT respectively. Significant difference in BMI were observed only in CET for 1^st^ and 3^rd^-4^th^, 2^nd^ and 3^rd^-4^th^, but only between 1^st^ and 4^th^ in 6MWT. Similarly, FFMI performance quartile gradation was significant for almost all quartiles but no significance was detected for the 6MWT (Figure 3). When performance quartiles were determined separately for men and women, men demonstrated more evident differences in FFMI quartiles compared to women. That fact indicates that FFMI may determine the exercise performance more in men than women with COPD (Please see online supplement for details, Additional file 1: Tables S1–S2; Additional file 2: Figures S1 & Additional file 3: Figure S2).

### Stepwise multiple linear regression

Reverse stepwise linear regression revealed that age, gender, FFMI, DLCO%, GOLD stage, Dyspnea and leg fatigue Borg scores at rest independently influence CET-T_end_ (Table 3). Age, BMI, FFMI, FEV_1_%, FVC/FEV_1_, GOLD stage, FRC, DLCO, and leg fatigue Borg score at rest were retained in the model when 6MWD was the dependent variable (Table 4). The model for CET was able to account for only 15.1% of the variability in cycle endurance time, while that for 6MWT accounted for 34.0% of the variability in 6MWD.

**Table 3:** Stepwise linear regression model of endurance cycle time in seconds

**r**^**2**^**: 15.1%**				
**Variable**	**Coefficient**	**Standard error**	**t value**	**p value**
Age, yrs	-3.35	0.57	-5.84	<0.001
Sex, m/f	-31.51	12.91	-2.44	0.015
FFMI, kg/m^2^	8.49	2.79	3.04	0.002
GOLD stage	-42.09	6.59	-6.39	<0.001
DLCO, %	1.21	0.32	3.83	<0.001
Dyspnea before test, index	-13.38	4.43	-3.02	0.003
Leg fatigue before test, index	-6.96	3.52	-1.98	0.048

Multiple linear regression model for parameters predictive of endurance cycle time

Using isolated linear regression determination a stepwise multiple linear regression model was established with an r^2^ of 15.1%.

**Table 4:** Stepwise linear regression model of Six minute walking distance in meters

**r**^**2**^**: 34.0%**				
**Variable**	**Coefficient**	**Standard error**	**t value**	**p value**
Age, yrs	-3.97	0.29	-13.98	<0.001
BMI, kg/m^2^	-7.48	0.75	-9.87	<0.001
FFMI, kg/m^2^	8.77	1.59	5.52	<0.001
FEV_1_, %	1.28	0.29	4.44	<0.001
FEV_1_/FVC, r	-1.60	0.42	-3.89	<0.001
GOLD stages	-15.04	5.04	-3.02	0.003
FRC, %	-0.43	0.11	-3.85	<0.001
DLCO, %	1.11	0.16	6.89	<0.001
Leg fatigue before test, index	-15.80	1.64	-9.57	<0.001

Multiple linear regression model for parameters predictive of six minute walk distance.

Using isolated linear regression determination a stepwise multiple linear regression model was established with an r^2^ of 34.0%.

## Discussion

In current study, we aimed to investigate the characteristics of functional exercise performance and the determinants which can influence the exercise performance in CET and the 6MWT in a large cohort of patients with moderate to severe COPD (Table 1). The main finding of this study is that CET-T_end_ can be determined by different physiological and clinical characteristics in comparison with the 6MWT. Of particular clinical importance, the sex of patients influenced CET-T_end_ but not in the 6MWD (Tables 2–4). Moreover, 6MWD seems to be more determined by lung function compared to CET-T_end_ (Tables 3–4). Also, a number of independent physiological and clinical characteristics demonstrated a different pattern in performance quartile gradation between CET and 6MWT (Figure 3).

Gender appears to be a strong determinant of CET-T_end_ in contrast to 6MWD. We found that men on average achieved a higher peak work rate and cycled longer during the CET than women; in spite of the fact the men were older with more impaired lung function (Tables 1 and 2). Previous studies have demonstrated that quadriceps muscle strength [22] and peak VO_2_[23,24] have small reductions in COPD patients over a 1 to 5 year period. Moreover, quadriceps muscle strength is positively related to DLCO in patients with COPD [25] and the studied male population had a greater diffusing capacity which could be accountable for the better cycle performance.

When we examined the performance quartiles, there was not as much of a gradation in gender proportion except the 4^th^ quartile among the 6MWD performance quartiles compared to that seen in the CET-T_end_ of performance quartiles. Furthermore, there was a significant difference between all the CET performance quartiles in FFMI, which was not seen in the 6MWD performance quartiles. However, as would be expected, FFMI was on average lower in the female population compared to men (Table 1). Thus, the question remains whether and to what extent gender differences in CET reflected the lower FFMI in women, or if the FFMI differences were simply a reflection of inherent gender differences. To examine this question we stratified the data into performance quartiles for each gender. We found that the difference in FFMI between performance quartiles was no longer apparent in women except for the 1^st^ and 4^th^ CET performance quartiles (Additional file 3: Figures S2), but still apparent in men between all the quartiles in CET and the 1^st^ and 4^th^, and the 1^st^ and 3^rd^ quartiles in the 6MWT. There was also less of a CET quartile gradation in FVC% and GOLD stage compared to that seen in 6MWD. To further elucidate the importance of gender and FFMI as determinants of test performance we carried out stepwise multiple regressions. The parameters that were retained when CET-T_end_ was used as the dependent variable were age, gender, FFMI, DLCO%, GOLD stage, Dyspnea and leg fatigue Borg scores at rest (Table 3). When 6MWD was used as the dependent variable, age, BMI, FFMI, FEV_1_%, FEV_1_/FVC, FRC%, DLCO%, GOLD stage and leg fatigue Borg score at rest were retained, but gender was not retained (Table 4). Performing a stepwise multiple regression after stratification by gender, FFMI was not retained in women but it was retained in men for the CET-Tend (Additional file 1: Tables S1-S2). Apparently quartile difference in FFMI (Figure 3) can be likely a reflection of an inherent gender differences beyond fat free mass. On Thus, it can be concluded that gender is an important determinant of CET-T_end_. Interestingly, peak aerobic capacity of patients with COPD was not determined by gender, after correction for isokinetic quadriceps peak torque, DLCO, FEV1, BMI, age and lower-limb lean muscle mass [26].

The model for 6MWD was much better at explaining variability in test performance (r^2^ of 34.0%) compared to that for CET (r^2^ of 15.1%). Other authors have also found it difficult to build a model that is highly predictive of CET performance [12,27]. This perhaps explains why we do not have a predictive equation for CET-T_end_ yet. It does however suggest that there are determinants of CET which we do not understand. This may reflect the complex nature of exercise intolerance in COPD.

CET-T_end_ seems to be influenced in a high extent from the degree of disease severity classified by GOLD stages more than the inter-individual variation of FEV_1_ or other specific clinical characteristics compared to 6MWT. It is observed that patients across the GOLD stages are characterized by increased physical deconditioning which leads to a progressive deterioration in lower limb muscle function [28]. The impact of the power/duration relationship on the exercise tolerance indicates that endurance is influenced by the intensity of exercise [29]. Both exercise tests are submaximal, however, the 6MWT is a self-paced while the CET at 75% is a constant load exercise test. The termination of exercise in CET is more associated to the inability to maintain the constant work load of exercise and thus CET-T_end_ is associated to an overall impaired health status, sex of patients and physical deconditioning more than specific lung function characteristics compared to the 6MWT.

In contrast to the findings of Neder and colleagues [10], we found that the vast majority of patients with COPD did not reach the 20 minute time point during a CET at 75% of peak work rate. This discrepancy between two studies is likely the result of differences in methodology and study populations. We retrospectively examined a clinical cohort of patients who were referred for pulmonary rehabilitation, whereas Neder and colleagues [10] studied a proscribed, smaller cohort with the intent of defining the power duration asymptote. In the present study, CET-T_end_ varied from 2 to 5 min for the most of the patients (Figure 1A). This is similar to the findings of Vivodtzev et al. [12] where most of the patients sustained CET for of 3 to 5 minutes. Although the proportion of patients with CET-T_end_ of 20 minutes was very small (2.5%), we evaluated the possibility of an underestimation in peak work rate that may result in unduly prolonged CET. Nevertheless, peak heart rate, ventilation and dyspnea scores following a cardiopulmonary exercise test indicated a maximum effort for those patients (Additional file 1: Table S3). Moreover, a quartile gradation of ventilation pattern derived by the maximum cardiopulmonary exercise test (CPET) in cycle ergometer indicates the effort to maintain O_2_ and CO_2_ homeostasis and may partly provide an insight of the ventilatory profile in patients of current study (Additional file 4: Figure S3).

Our findings showed a large variation in the CET-T_end_ values (Figure 1A) which is also observed in the 6MWD, though to a lesser extent (Figure 1B). Therefore, evaluating patients with a CET at 75% of peak work rate leaves room for demonstrating improvement with an intervention, especially considering that the minimal clinically important difference has been reported to be between 100 and 200 seconds [9] or ~33% increased from baseline [4]. Although the identical exercise intensity of 75% of peak work rate, the physiological stress may differ amongst patients and thus the space for improvement through PR exercise modalities can vary. Moreover, in daily life the majority of patients have been adapted to walking but not to cycling exercise and that may results in a greater potential of improvement in CET compared to 6MWT after PR. Nevertheless, O’Donnell et al. [15] demonstrated that CET at 75% peak work rate was highly repeatable across run-in visits: 7.9+/-4.8 and 8.4+/-5.1 min (R = 0.84) in 463 COPD patients indicating the appropriateness of CET in the exercise assessment of patients with COPD.

There are methodological considerations to our study. It is a retrospective cohort study which means there may be unidentified confounders. We have no way of knowing why some of the records were incomplete. Surely some may be incomplete due to a failure of the patients to complete the initial pulmonary rehabilitation assessment, or refusal to perform a particular test. Some were also repeat evaluations. Nonetheless the large sample size helps to mitigate some of these potentially confounding factors. Secondly, this is a population of patients who have been referred for pulmonary rehabilitation which could result in a referral bias towards patients with a greater degree of limitation. Regardless, our findings sufficiently demonstrate that performing CET at 75% of peak work rate is a reasonable effort level for a population of COPD patients being referred for pulmonary rehabilitation. However, a fixed proportion of peak work rate exercise does not provide identical physiological stress amongst patients. Furthermore, this study increases our understanding of gender differences in exercise testing and highlights the complex pathophysiology of exercise intolerance in COPD. There have been a number of gender differences reported in COPD [27,30-36]. Most intriguingly, it has previously been shown that women have higher symptom scores on the St. George’s Respiratory Questionnaire (SGRQ) compared to men [27]. It may therefore be that compared to 6MWT, which simulates a more routine daily activity, cycle ergometry evokes more symptoms in women compared to men resulting in reduced CET-T_end_ for women. This could be a reflection of the fact that most patients with COPD, even in the Netherlands, do not routinely ride a bicycle, and thereby cycling may evoke more dyspnea because of its unfamiliarity. In lieu of predictive equations, this gender difference in CET should be taken into consideration and used to temper the interpretation of CET. In summary, CET at 75% of peak work rate appears to be a useful clinical exercise tool in COPD. There is plenty of room to demonstrate improvement with an intervention and it measures a different aspect of exercise tolerance compared to 6MWT. The fact that the determinants of CET-T_end_ are not reducible to a model built from a handful of clinical parameters also speaks to the importance of CET-T_end_ as an independent measurement before and after pulmonary rehabilitation. Furthermore, CET reflects gender differences in COPD, an aspect of the disease that is gaining increasing recognition.

## Conclusion

CET and 6MWT are not interchangeable exercise tests. CET-T_end_ is a valuable outcome of CET as it is related to multiple clinical aspects of disease severity in COPD. Gender difference should temper the interpretation of CET. BMI, FEV_1_ and FRC influence the walking performance of 6MWT rather than the CET. Although the fixed proportion of workload capacity does not provide the same physiological stress among patients, improvement in CET-T_end_ after pulmonary rehabilitation can reflect a total improvement of clinical status in patients with COPD.

## Abbreviations

BMI: Body mass index; COPD: Chronic Obstructive Pulmonary Disease; CET: Cycle endurance test; CET-T_end_: Cycle endurance time of duration; DLCO: Diffusion capacity for carbon monoxide; FEV_1_/FVC: The ratio (r) of forced expiratory volume in 1 second and forced vital capacity; FEV_1_: Force expiratory volume in 1 second; FFMI: Fat free mass index; FRC: Functional residual capacity; FVC: Forced vital capacity; MRC: Medical Research Council dyspnea scale; PaCO_2_: Partial arterial pressure of carbon dioxide; PaO_2_: Partial arterial pressure of oxygen; PR: Pulmonary Rehabilitation; SpO_2_: Saturation of peripheral oxygen; Watt _absolute values_: Absolute peak work rate achieved on maximal incremental cycle test; Watt_%predicted_: Percent of predicted peak work rate achieved on maximal incremental cycle test; 6MWD: Six-minute walk distance; 6MWT: Six-minute walk test.

## Competing interest

All authors declare not having competing interests that might have influenced the performance or presentation of our work in this manuscript.

## Authors’ contribution

VA contributed to data acquisition, data and statistical analysis, figures design and drafted the manuscript. SW contributed to the data acquisition, drafted the manuscript. MG contributed to the data acquisition, statistical analysis and critically reviewed the manuscript. LV contributed to the patients’ medical assessment, medical surveillance during exercise tests and critically reviewed the manuscript. FF contributed to the patients’ medical assessment, medical surveillance during exercise tests and critically reviewed the manuscript. FS contributed to the writing of the manuscript and critically reviewed the manuscript. IV contributed to the writing of the manuscript and critically reviewed the manuscript. EW contributed to the writing of the manuscript and critically reviewed the manuscript. MS contributed to the writing of the manuscript, critically reviewed and revised the manuscript. All authors read and approved the final manuscript.

## Pre-publication history

The pre-publication history for this paper can be accessed here:

http://www.biomedcentral.com/1471-2466/14/97/prepub

## Supplementary Material

Additional file 1 Supplementary information. Click here for file

Additional file 2 **Performance quartile comparisons of men.** Using the frequency distribution 1134 male patients were separated into performance quartiles. With the quartiles increasing left to right (CET: quartiles with wave hashing, 6MWT: quartiles with diagonal hashing). Comparisons were then made between the quartiles of CET and between quartiles of 6MWT for all variables. Significant differences are demonstrated by lines above the relevant bars. Click here for file

Additional file 3 **Performance quartile comparisons of women.** Using the frequency distribution 868 female patients were separated into performance quartiles. With the quartiles increasing left to right (CET: quartiles with wave hashing, 6MWT: quartiles with diagonal hashing). Comparisons were then made between the quartiles of CET and between quartiles of 6MWT for all variables. Significant differences are demonstrated by lines above the relevant bars. Click here for file

Additional file 4 **Performance quartile comparisons of CPET.** Using the frequency distribution patients were separated into performance quartiles. With the quartiles increasing left to right (Quartiles with wave hashing for all sample, males and females). Comparisons were then made between the quartiles of CPET in cycle ergometer for the achieved maximum ventilation (VEmax) expressed as absolute values and percentage of predicted. Significant differences are demonstrated by lines above the relevant bars. Click here for file
