# Supplementary material for: Characteristics and determinants of endurance cycle ergometry and six-minute walk distance in patients with COPD
Source: BMC Pulm Med. 2014 May 31;14:97. doi: 10.1186/1471-2466-14-97 (PMC4229855; doi:10.1186/1471-2466-14-97)
Supplement: Additional file 1 — Supplementary information. [file 1471-2466-14-97-S1.docx]

**SUPPLEMENTARY INFORMATION**

**Supplementary Tables**

Table S1

| Table S1. Stepwise linear regression model of CET-T_end_ | | | | | |
| --- | --- | --- | --- | --- | --- |
| ♂ **MALES** |  |  | |  | r^2^: 17.2% |
| **Variable** | **Coefficient** | **Standard Error** | | **t value** | **p value** |
| Age, yrs | -2.74 | 0.78 | | -3.52 | <0.001 |
| FFMI, kg/m^2^ | 13.00 | 3.56 | | 3.65 | <0.001 |
| GOLD stage | -44.24 | 8.91 | | -4.97 | <0.001 |
| DLCO, % | 1.25 | 0.42 | | 2.98 | 0.003 |
| Dyspnea before test, index | -21.9 | 4.73 | | -4.64 | <0.001 |
|  | | | | | |
| ♀ **FEMALES** | | | | | r^2^: 11.0 % |
| **Variable** | **Coefficient** | | **Standard Error** | **t value** | **p value** |
| Age | -3.99 | | 0.84 | -4.74 | <0.001 |
| FEV_1_, % | 1.79 | | 0.44 | 4.04 | <0.001 |
| DLCO, % | 1.06 | | 0.47 | 2.26 | 0.024 |
| Leg fatigue before test, index | -12.01 | | 4.91 | -2.45 | 0.015 |

Table S1: Multiple linear regression model for parameters predictive of CET-T_end_  (in seconds) in males and females.

Table S2

| Table S2. Stepwise linear regression model of Six minute walking distance in meters | | | | | |
| --- | --- | --- | --- | --- | --- |
| ♂ **MALES** |  |  | |  | r^2^: 32.5% |
| **Variable** | **Coefficient** | **Standard Error** | | **t value** | **p value** |
| Age, yrs | -3.79 | 0.41 | | -9.22 | <0.001 |
| BMI, kg/m^2^ | -3.46 | 0.81 | | -4.27 | <0.001 |
| FEV_1_, % | 1.72 | 0.42 | | 4.11 | <0.001 |
| FEV_1_/FVC, % | -1.25 | 0.55 | | -2.26 | 0.024 |
| GOLD stage | -16.39 | 7.36 | | -2.23 | 0.026 |
| DLCO, % | 1.26 | 0.22 | | 5.77 | <0.001 |
| Leg fatigue before test, index | -19.28 | 2.32 | | -8.31 | <0.001 |
|  | | | | | |
| ♀ **FEMALES** | | | | | r^2^: 35.9% |
| **Variable** | **Coefficient** | | **Standard Error** | **t value** | **p value** |
| Age | -4.06 | | 0.39 | -10.43 | <0.001 |
| BMI, kg/m^2^ | -4.92 | | 0.69 | -7.19 | <0.001 |
| FEV_1_, % | 0.69 | | 0.30 | 2.28 | 0.023 |
| GOLD stage | -16.04 | | 6.61 | -2.43 | 0.016 |
| DLCO, % | 1.01 | | 0.22 | 4.50 | <0.001 |
| Leg fatigue before test, index | -12.61 | | 2.27 | -5.55 | <0.001 |

Table S2: Multiple linear regression model for parameters predictive of 6MWD (in meters) in males and females.

Table S3

| Table S3. Evidences of maximum CPET in 51 patients with CET ≥20min | | |
| --- | --- | --- |
| **HRmax ≥80% predicted**  **↓**  N= 31 (61%) patients | **VEmax ≥85% MVV**  **↓**  N= 18 (35%) patients | **Dyspnea and/or leg fatigue ≥7**  **↓**  N= 43 (84%) patients |
| **Of rest patients:** N= 20 (39%)  ● 1 (2%) had only VEmax  ≥85% MVV  ● 12 (23%) had only dyspnea and/or leg fatigue ≥7 by BORG  ● 7 (14%) had both VEmax 85% MVV & dyspnea and/or leg fatigue by BORG scale | **Of rest patients:** N= 33 (65%)  ● 5 (10%) had only HRmax ≥80%pred.  ● 13 (26%) had only dyspnea and/or leg fatigue ≥7 by BORG  ● 15 (29%) had both HRmax ≥80%pred. & dyspnea and/or leg fatigue by BORG scale | **Of rest patients:** N= 8 (16%)  ● 1 (2%) had only VEmax  ≥85% MVV  ● 5 (10%) had only HRmax ≥80%pred.  ● 2 (4%) had both VEmax ≥85% MVV & HRmax ≥80%predicted |

Table S3: Percentages of patients who reached a high threshold value in heart rate (HRmax) and/or ventilation (VEmax) and/or Borg scores (Dyspnea/Leg Fatigue) as evidence of an achieved maximum effort during the maximum cardiopulmonary exercise test (CPET) in cycle ergometer.
