# Supplementary figures and images for: Characteristics and determinants of endurance cycle ergometry and six-minute walk distance in patients with COPD
Source: BMC Pulm Med. 2014 May 31;14:97. doi: 10.1186/1471-2466-14-97 (PMC4229855; doi:10.1186/1471-2466-14-97)

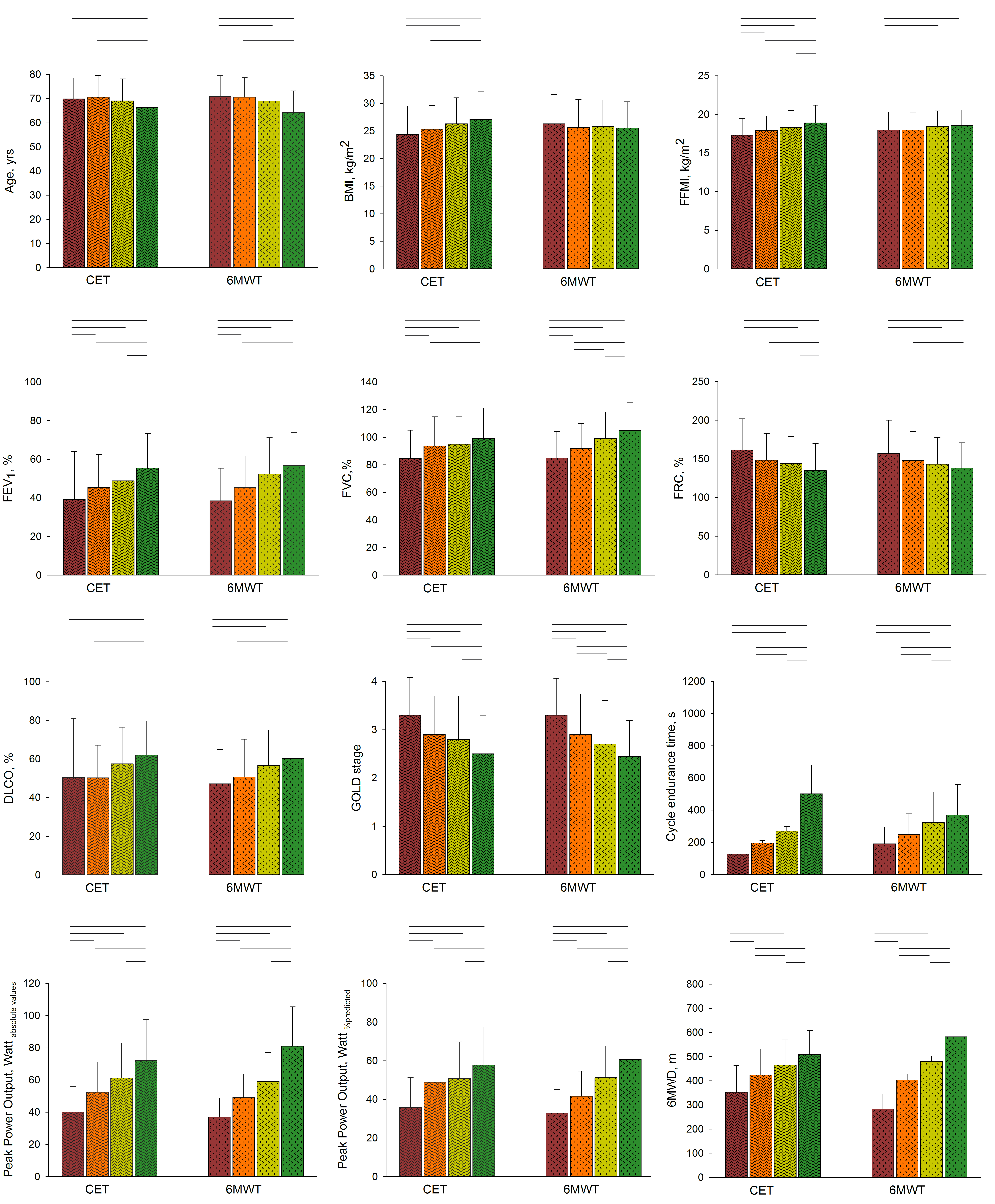

Supplement: Additional file 2 — Performance quartile comparisons of men. Using the frequency distribution 1134 male patients were separated into performance quartiles. With the quartiles increasing left to right (CET: quartiles with wave hashing, 6MWT: quartiles with diagonal hashing). Comparisons were then made between the quartiles of CET and between quartiles of 6MWT for all variables. Significant differences are demonstrated by lines above the relevant bars. [file 1471-2466-14-97-S2.tiff]

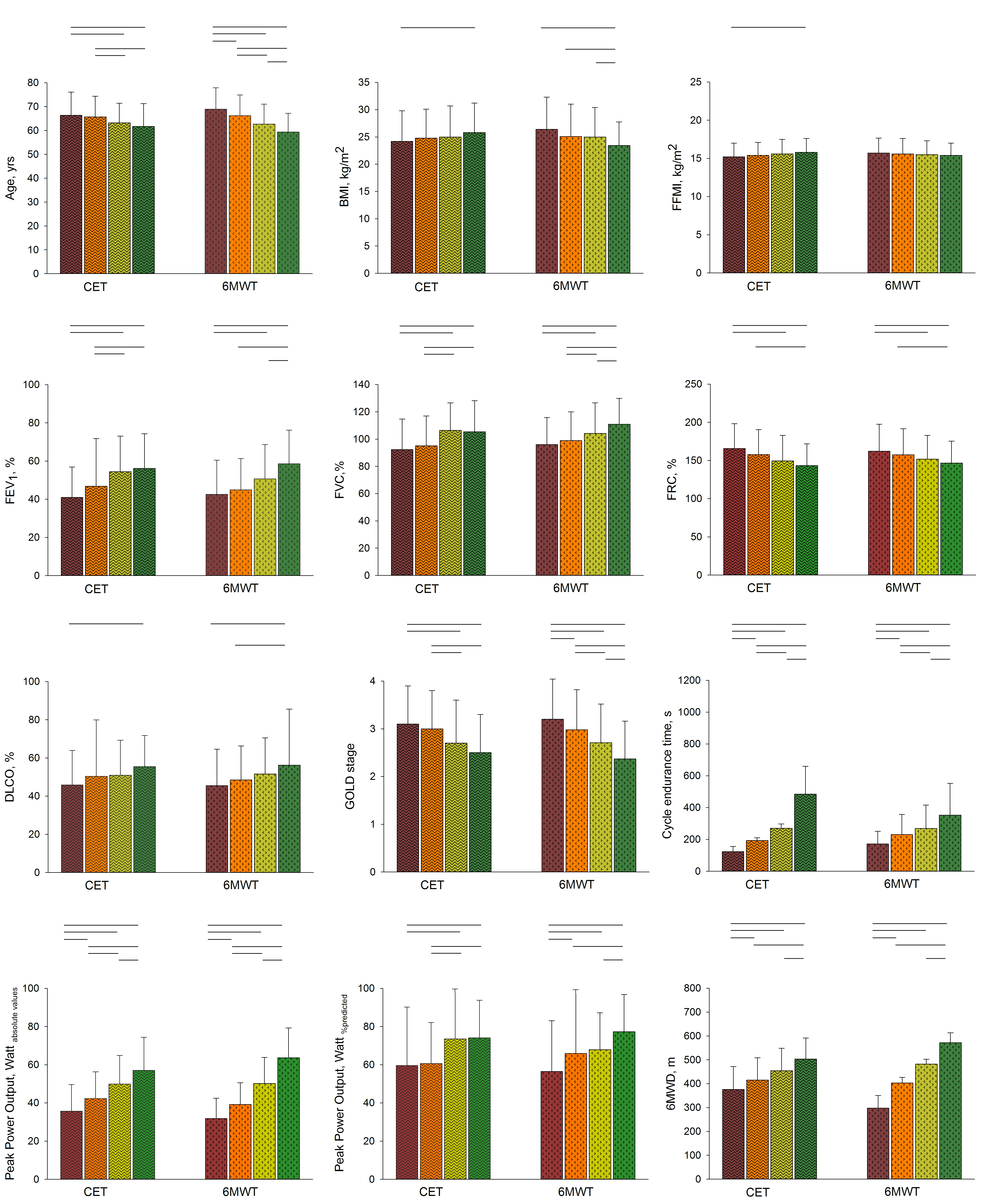

Supplement: Additional file 3 — Performance quartile comparisons of women. Using the frequency distribution 868 female patients were separated into performance quartiles. With the quartiles increasing left to right (CET: quartiles with wave hashing, 6MWT: quartiles with diagonal hashing). Comparisons were then made between the quartiles of CET and between quartiles of 6MWT for all variables. Significant differences are demonstrated by lines above the relevant bars. [file 1471-2466-14-97-S3.tiff]

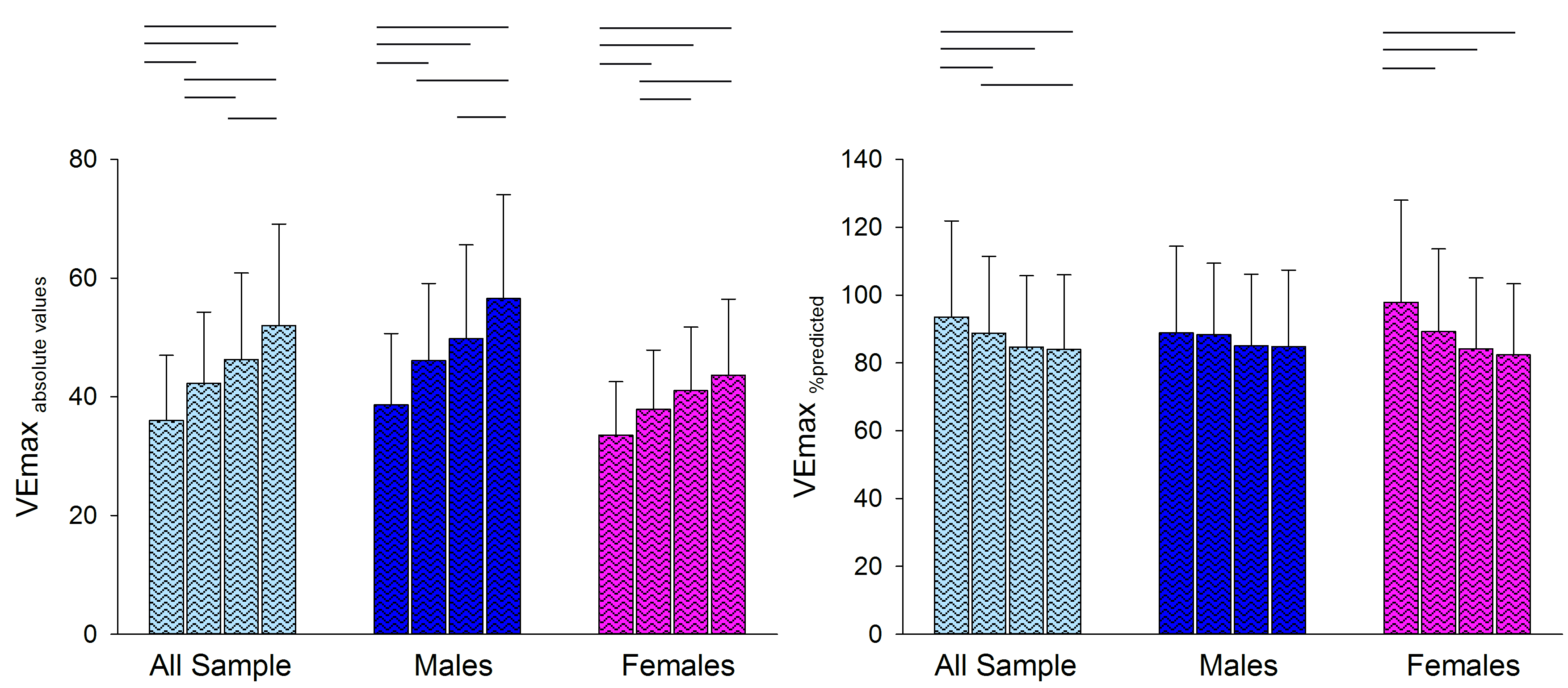

Supplement: Additional file 4 — Performance quartile comparisons of CPET. Using the frequency distribution patients were separated into performance quartiles. With the quartiles increasing left to right (Quartiles with wave hashing for all sample, males and females). Comparisons were then made between the quartiles of CPET in cycle ergometer for the achieved maximum ventilation (VEmax) expressed as absolute values and percentage of predicted. Significant differences are demonstrated by lines above the relevant bars. [file 1471-2466-14-97-S4.tiff]
